# Gastritis and Gastric Ulcers in Working Dogs

**DOI:** 10.3389/fvets.2016.00030

**Published:** 2016-04-04

**Authors:** Michael S. Davis, Katherine K. Williamson

**Affiliations:** ^1^Department of Physiological Sciences, Center for Veterinary Health Sciences, Oklahoma State University, Stillwater, OK, USA; ^2^Purina Animal Nutrition, Land O’Lakes Inc, Gray Summit, MO, USA

**Keywords:** exercise, stomach ulcers, gastritis, sled dogs, hyperthermia, omeprazole, working dogs, retrievers

## Abstract

Gastritis and gastric ulcers are an important cause of morbidity and mortality in canine athletes. Although the majority of scientific work on this condition has been performed in ultraendurance racing sled dogs, this condition has been identified in other canine athletes, including sled dogs competing in shorter events and dogs performing off-leash explosive detection duties. The cause of the syndrome is unknown, but current hypotheses propose a link between exercise-induced hyperthermia and loss of gastric mucosal barrier function as an early event in the pathogenesis. Treatment is focused on prevention of clinical disease using acid secretion inhibitors, such as omeprazole, which has excellent efficacy in controlled clinical studies.

## Introduction

Gastritis and gastric ulcers can be an important cause of morbidity and mortality in canine athletes, most notably racing sled dogs but also other athletic dog populations. This review will outline the important points regarding this condition, including prevalence and risk factors, clinical syndrome, pathophysiology, and treatment and prevention strategies.

## Prevalence and Importance

The importance of exercise-induced gastric disease (EIGD) in dogs is highlighted by both the frequency with which this syndrome is directly or indirectly related to mortality, but also the more insidious effect on performance and overall thriftiness. For the past two decades, organized ultraendurance sled dog racing has required the meticulous documentation of sled dog deaths in an attempt to provide transparency to the fans and critics of the sport as well as identify areas of canine health and well-being that require additional investigation. These reports showed that from 1994 to 2006, 23 dogs died during the 13 Iditarod races held during that time span (1). Eleven of those deaths were either directly or indirectly related to gastric disease (blood loss or vomiting and aspiration of gastric contents, respectively). Comprehensively collated statistics such as these are not available for other major races, but anecdotal evidence supports a similar proportional pattern (albeit with smaller numbers overall due to the fewer numbers of dogs involved in these other events). These statistics do not reflect the unknown number of dogs that may have been affected in a less severe manner, and were dropped off at checkpoints along the racecourse. Strenuous exercise requires the consumption of considerable amounts of food and water – ultraendurance racing sled dogs will burn from 8000 to 12000 kcal/day (2, 3) and turnover 5 l of bodywater/day (4). Any illness that reduces a dog’s appetite or makes them reluctant to eat or drink or promotes vomiting/regurgitation – as gastric disease is known to do – will rapidly cause poor performance and dehydration under these conditions.

Some of the earliest studies of the prevalence of gastric disease in athletic dogs were done in association with the Iditarod Sled Dog race. In 2000, a small pilot study was performed to follow-up on anecdotal work done by Drs. Jack Morris and Phil Meyer, in which they reported frequently finding gastric lesions in dogs following the race. The 2000 study was conducted on dogs returning from the race, and found a gastric lesion prevalence of approximately 35% in dogs that were examined from 3 to 7 days post-exercise (5). Some of these subjects had completed the race, but most had been “dropped” for various medical reasons (not always due to suspected gastrointestinal disease). The first systematic evaluation of gastric health in racing sled dogs was performed the following year. Gastric endoscopy was performed on 73 dogs within 24 h of finishing the race (5). Using the visible presence of at least one area of erosion or ulceration in the gastric mucosa as the criterion, nearly half of the dogs had endoscopically visible lesions that were considered clinically significant. This percentage has held up through seven different studies: unmedicated racing sled dogs can be expected to have between 50 and 70% prevalence of clinically significant lesions after at least a single day of exercise (6), whether it is a long training day (7), a mid-distance race (8), or one of the ultraendurance races (5, 9–11). It seems intuitive that exercise intensity has some influence on disease severity, but further discussion of this type requires more careful definition of “exercise intensity,” which can be quantified many different ways. Within the scope of a 1000+-mile race, finishing place does not seem to have a major influence on prevalence – teams finishing in 12 days (averaging 83 miles/day) had similar prevalence values to teams finishing in 9 days (averaging 111 miles/day) (5). Although data are not available from this study to assess whether the difference in daily distance was due to higher speeds or shorter rests in the teams finishing in 9 days, in general the lower-placing teams do so by resting longer as well as traveling slower. Teams competing in mid-distance races averaging 150 miles/day, during which substantially less rest/day is taken compared to the longer distance races, had noticeably higher prevalence and average gastric endoscopy severity scores (ESSs), suggesting that intensity of exercise (or something that is strongly correlated to intensity of exercise) has an influence on EIGD (8).

There have been studies of other athletic dog populations that have helped clarify the scope of this issue in the world of canine athletes. A study of field trial retrievers participating in a simulated single-day competition found a statistically significant worsening of the gastric endoscopy scores, but the scores stayed within the range that is considered not clinically significant (12). Thus, this population is probably not at risk for exercise-induced gastritis/gastric ulcers as a primary disease process, but consideration should be given to taking preventive measures if an individual competitive dog has additional risk factors for gastric disease. On the other hand, retrievers used for off-leash explosive detection work (during which the dogs may be exercising intermittently for up to 9 h/day) had an 84% prevalence of clinically severe gastric ulcers after five consecutive days of exercise, with the mean endoscopy score of the exercising dogs being higher than any other group of exercising dogs examined in other studies (13). Thus, it is clear that dogs other than racing sled dogs are at risk for EIGD if they perform sufficiently strenuous exercise.

## Definition of Clinical Syndrome

Perhaps the most frustrating aspect to this disease is that the overwhelming majority of affected dogs are subclinical, as evidenced by the fact that all endoscopy studies to date have been done in dogs that were clinically normal – having completed a grueling competition and/or having passed the physical examination that preceded the general anesthesia for endoscopy (5–11, 13). At the opposite end of this clinical spectrum are the dogs that are withdrawn from competition due to unequivocal evidence of gastric disease (i.e., repeated vomition or vomiting of fresh blood, aspiration pneumonia), but these dogs are relatively rare. The proportion of dogs that are withdrawn from competition that may have EIGD as an underlying cause (for example, due to lack of appetite or dehydration) is unknown, but it is likely that these “dropped” dogs have a prevalence at least as high as those dogs that continue to perform well.

There are no clinical pathology measurements that have been shown to characterize EIGD reliably. With the exception of the relatively rare instance in which the disease progresses to severe, acute, or chronic blood loss, decreased erythrocyte and serum protein concentrations are common in dogs performing endurance exercise (14), but not consistently associated with EIGD. Hematocrit may decrease in response to plasma volume expansion, which is a known and predictable conditioning response (15). Thus, differentiating mild hemodilution secondary to a desirable conditioning effect from occult moderate blood loss can be challenging in the athletic dog population. Serum cortisol predictably increases in exercise challenged dogs (16–19), likely due to the need for gluconeogenesis to offset the increased demand for glucose as a metabolic fuel (20), thus decreasing the usefulness of assessing serum cortisol as an indicator of physiological stress that could predispose to EIGD. In the studies in which this marker has been reported in the same dogs for which gastric examinations were performed, there was no association between serum cortisol and gastric disease on an individual dog basis (even though there was concurrent increase in serum cortisol and gastric disease in athletic dogs as a group) (11), suggesting that the increases in serum cortisol were not related either causally or as an effect of EIGD.

**Figure 1:**
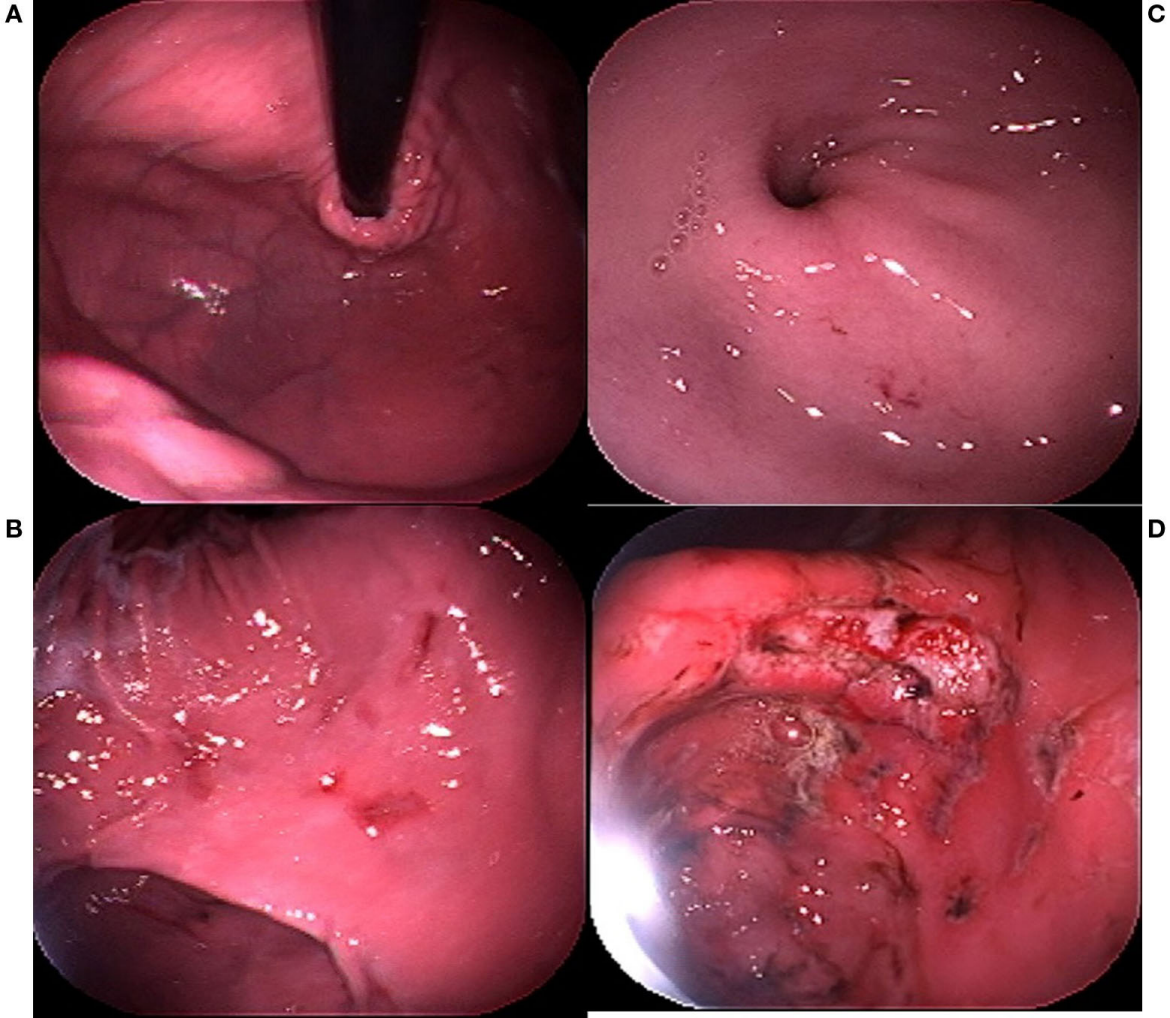
**Endoscopy severity score (ESS) guide**. **(A)** ESS = 0; **(B)** ESS = 1; **(C)** ESS = 2; **(D)** ESS = 3.

Endoscopy remains the gold standard for the diagnosis of EIGD in dogs due to the lack of consistent clinical signs or laboratory indices in the majority of the affected dogs. The range of severity of endoscopic findings can be substantial, from a few submucosal petechia to multiple actively bleeding lesions. A subjective severity scoring system has been established in which a stomach completely free of visible lesions is scored as 0; a stomach with a few submucosal petechia but no visible defects in the mucosa is scored as 1, a dog with extensive areas of erosions OR a single bleeding ulcer is scored as 2, and a dog with multiple bleeding ulcers is scored as 3 (Figure 1). Lesions can be found in all regions of the stomach, and through hundreds of examinations a particular bias in lesion location within the stomach has not been identified. Endoscopic assessment of motility, while subjective, is noteworthy in that as opposed to impaired motility (which can be associated with gastric lesions), the typical athletic dog appears to have enhanced gastrointestinal motility. The stomach of a trained working dog routinely can be examined, free of ingesta, after only a 12-h fast. Though of minimal scientific value, this observation may have considerable logistical value in deciding whether to perform gastric endoscopy on a dog with suspected EIGD.

Histopathology of dogs affected with EIGD has provided important clues regarding the pathophysiology of the disease. As a general practice, biopsies are obtained from normal-appearing areas of the gastric mucosa to avoid the secondary inflammation and necrosis that is expected in areas of actively bleeding lesions. Nevertheless, these biopsies have consistently demonstrated chronic mononuclear inflammation (5, 21), suggesting that although the gross lesions may have appeared in <24 h, the underlying pathological process has been occurring much longer – often predating the exercise session that appears to have caused the gastric mucosal disruption. Of particular interest is histopathological evidence of extensive apoptosis of grossly normal mucosa, suggesting that the terminal event in the pathophysiology is the programed cell death and sloughing of the mucosal epithelium, rather than chemical erosion and damage of the cells by stomach acid. Evidence of chronic mononuclear inflammation is present even in dogs that have been rested for up to 4 months (21). This type of chronic inflammation is not present in field trial retrievers (12), supporting both the contention that this population of dogs is not affected routinely by EIGD and the possible role of chronic mononuclear inflammation in the development of EIGD.

## Pathophysiology

There are few studies that specifically address the mechanisms underlying the development of EIGD in dogs. Increased permeability of the gastrointestinal mucosa has been measured in response to exercise in racing sled dogs (6, 10, 11). Although it is impossible to determine whether the loss of barrier function measured in these studies reflects an early pathophysiological event or is the result of the disease, it is worthwhile to note that the magnitude of increase in serum sucrose (a marker of permeability of the gastric mucosa) did not correlate with the endoscopically assessed severity of gastric mucosal disruption. With increased paracellular permeability, virtually anything that is in the lumen can find its way into the walls of the respective organs. In the stomach, the primary noxious material for diffusion is acid. With chronic, repeated periods of hyperthermia, the intermittent paracellular acid leak not only causes chronic subclinical inflammation (demonstrated in clinically normal, fully trained sled dogs prior to competitive exercise) but also sets the stage for acute exacerbation of the disease on the first day of competition.

The possibility of increased mucosal permeability as an early event is supported by studies in rodents and humans, demonstrating that physiological hyperthermia comparable to the magnitude routinely documented in exercising dogs will result in increased transmucosal permeability of other segments of the gastrointestinal tract (22–24). A primary role of hyperthermia-induced loss of gastrointestinal mucosal barrier function is further supported by the consistency between disease severity and the time-weighted duration of hyperthermia when all studies are considered: explosive detection dogs will achieve and sustain remarkably high rectal temperatures during simulated deployment activities (13), higher than is routinely found in racing sled dogs. In turn, racing sled dogs that are participating in relatively shorter distance events (300 miles) will tend to maintain higher speeds and rest less often than dogs competing in the ultraendurance events of 1000 miles. Finally, training runs tend to be slower and shorter in duration than racing, presumably resulting in less hyperthermia. Although this correlation is admittedly crude, its general fidelity with the population-wide variations in gastric disease severity, combined with the mechanistic data available in other species, makes hyperthermia-induced mucosal permeability a leading candidate for the initiating step in this syndrome.

The effects of “stress” are often cited as the cause of gastric ulcers in a variety of animals and circumstances. Commonly, physiological stress is quantified through the measurement of serum or salivary cortisol – an approach that may be too simplistic to provide a clear explanation or mechanistic basis. Glucocorticoids have a very wide range of biological activities, including the upregulation of hepatic gluconeogenesis (20), so it is not surprising that increases in serum cortisol are measured routinely during and following strenuous exercise (16–19) when the rate of glucose oxidation by working muscle greatly increases (25). Although the association between large doses of exogenous corticosteroids and gastric ulcers has been documented (26), a similar association between endogenous corticosteroids and gastric disease is more problematic for the simple reason that when investigators document concurrent hypercortisolemia and gastric disease, it is impossible to determine whether the increased release of cortisol is causing the disease or a response to the disease (or both). Similarly, in exercising animals – particularly those with modest increases in serum cortisol that are consistent with the requirement for increased glucose availability, it is difficult to make a firm association between hypercortisolemia and gastric disease. Indeed, in the single study reporting concurrent measurements, there was not an individual association between the magnitude of gastric disease and serum cortisol concentration (11). Thus, the role of glucocorticoids in the pathophysiology of exercise-induced gastrointestinal disease remains unconfirmed.

Increased secretion of gastrin (a potent gastric acid secretagogue) has been documented in both human (27–29) and equine athletes (30), and is believed to contribute to EIGD in these species through increased gastric acidity. Although a potential role for gastric acid in the pathogenesis of EIGD in dogs can be suggested due to the documented protective effects of acid suppressing medications, neither increased gastrin nor gastric hyperacidity has been documented in athletic dogs at this time. Therefore, the potential role for increased gastrin secretion in the pathogenesis of EIGD remains possible, but speculative.

There are many proposed causes of EIGD in dogs, but none that have been definitively proven at this time. Many, however, can be regarded as unlikely as major contributing factors to the disease in general. For example, the distribution of lesions observed during gastroscopy (widely distributed with no particular region predominantly affected) would tend to discount the possibilities of direct tissue trauma such as might occur when sled dogs are fed frozen snacks (5). The possibility of trauma secondary to ingestion of frozen food can be further disregarded in light of the prevalence of gastric lesions in studies in which the dietary intake of the dogs was closely controlled (8, 13). The fact that severe lesions have been found in individual dogs in one year and virtually nothing the following year (or vice versa) tends to reduce the likelihood of individual susceptibility as a major contributing factor. While the possibility that a high-fat diet may contribute to susceptibility cannot be discounted (since virtually all athletic dogs are consuming a high-fat diet in order to match energy intake with expenditure, even the military explosive detection dogs), minor variations in diet composition within that broad category of “high energy” diets do not seem to have a major influence on disease prevalence. Whether the high-fat diets routinely used in these types of canine athletes predispose to EIGD has not been examined. Gastric pathogens, such as *Helicobacter pylori*, are a major cause of gastric disease in humans and, therefore, the possibility of these organisms as a contributing cause to canine EIGD has been investigated. Special stains on biopsies obtained in three different studies have failed to demonstrate consistently the presence of helical bacteria, and in the instances in which these genera were detected, there was no association with either visible or histopathological evidence of disease (5, 21). Finally, exercise-induced ischemia due to the redistribution of visceral blood flow is possible in dogs for which the intensity of exercise exceeds the capacity of the cardiovascular system. However, direct measurements of visceral blood flow in trained sled dogs exercising at typical ultraendurance racing speeds found no reduction in splanchnic blood flow (31), suggesting that at least in these populations (as well as populations exercising at a similar intensity or lower such as explosive detection dogs), splanchnic ischemia is not a major factor in the development of EIGD.

Non-steroidal anti-inflammatory drugs (NSAIDs) deserve special mention due to their well-known ulcerogenic potential (32–34). Although it is possible that concurrent administration of NSAIDs could have an additive or synergistic role in promoting gastric disease in exercising dogs, it should be noted that all of the studies to date that have demonstrated EIGD in dogs have been done in circumstances in which NSAID use was prohibited [either in organized competition in which random drug testing is a prominent feature (5, 8–10) or in controlled studies in which the investigators could exclude the use of NSAIDs (6–8, 13)]. Thus, it can be safely concluded that NSAIDs are not a necessary component of the pathophysiology of EIGD in dogs.

## Treatment and Prevention

The treatment of EIGD in dogs is not dissimilar to the treatment of gastric ulcers of any cause, with the sole exception of cessation of the inciting cause (exercise) being a prominent aspect of EIGD treatment. Acid suppression is the most important component in the treatment and prevention of gastric ulcers. There are two main drug classes currently in use in veterinary medicine for acid suppression. Histamine-2 (H2) receptor antagonists, such as cimetidine, ranitidine and famotidine, act to suppress gastric acid secretion by binding the H2 receptors of gastric parietal cells, thus, preventing the secretion of both hydrochloric acid and pepsin. Proton pump inhibitors, such as omeprazole, act to block the H^+^–K^+^ATPase proton pump that is the final stage of gastric acid secretion. While proton pump inhibitors are considerably more potent antacids than H2 blockers, the realities of dosing should be considered in drug selection. Unlike proton pump inhibitors, H2 antagonists have good oral bioavailability when administered with food, and, therefore, need not be administered on an empty stomach as is the case with omeprazole. This fact is significant when one considers the difficulties of manual oral administration to dogs in extreme conditions or situations, and that frequent meals are required to meet the caloric demands of heavily exercising dogs making administration on an empty stomach problematic. To further illustrate this point, the efficacy of omeprazole in preventing gastric ulcers in sled dogs under racing conditions was the first preventive therapy investigated by Davis et al. (9), and the results were disappointing, showing that omeprazole was only moderately effective in preventing clinically significant gastric ulcers in dogs finishing the Iditarod Sled Dog race. Further unpublished research later determined that the absorption of proton pump inhibitors administered with a meal to dogs was extremely poor. With this in mind, the authors undertook a series of studies to determine the effectiveness of famotidine (which is well absorbed in the presence of food) in preventing gastric ulcers in racing sled dogs. The initial study indicated that under relatively modest conditions (training runs up to 100 miles in a day), famotidine was effective in preventing gastric ulcers (7). However, a subsequent study showed that, under actual racing conditions, famotidine was not sufficiently effective in preventing severe EIGD (8). A further study was then conducted, which compared the efficacy high-dose famotidine (40 mg PO BID/~25 kg dog) with omeprazole (20 mg PO SID/~25 kg dog) in preventing EIGD under racing conditions. This study showed that, with carefully timed administration, near the conclusion of a long exercise bout during which minimal snacking has occurred, omeprazole is more effective in reducing the number and severity of gastric lesions in racing sled dogs than famotidine (8). If an additional 30 min is allowed to pass prior to feeding the dog, efficacy can approach 100% in preventing clinically significant lesions during even the most strenuous exercise events.

Sucralfate, a GI protectant that binds to proteinaceous exudates found at ulcer sites, should be administered to dogs with confirmed or suspected gastric ulcers. The primary benefit of sucralfate is that it protects the ulcerated areas from further damage from gastric acid, bile, and pepsin. It has also been shown to have cytoprotective and antacid effects. Supportive care may include ensuring adequate hydration and nutrition, and transfusions in the case of excessive blood loss. Specific treatment for sequelae such as aspiration pneumonia may be necessary. Canine athletes, like any athlete, occasionally suffer from musculoskeletal injury for which NSAIDs are indicated. The potential contribution of NSAIDs to EIGD is unknown, but given the ulcerogenic potential of NSAIDs, these drugs should not be used before or during the types of exercise known or suspected to predispose a dog to the development of EIGD. NSAIDs should be used judiciously in dogs who are at risk of or suspected to have EIGD and concurrent acid suppression therapy should be considered.

## Author Contributions

All authors listed, have made substantial, direct and intellectual contribution to the work, and approved it for publication.

## Conflict of Interest Statement

The authors declare that the research was conducted in the absence of any commercial or financial relationships that could be construed as a potential conflict of interest.
